# BREATHE: The Health Data Research Hub for Respiratory Health.

**DOI:** 10.23889/ijpds.v7i3.1995

**Published:** 2022-08-25

**Authors:** Elizabeth Nelson, Frances Burns

**Affiliations:** 1 Administrative Data Research Centre Northern Ireland (ADRC NI); 2 Northern Ireland Trusted Research Environment (NITRE)

There is an increasing awareness of the potential for creating benefit through the production, use and linkage of data sets to enhance evidence for public policymaking, service provision and the general public good. By linking large scale datasets across thematic areas, researchers are able to identify trends and areas of concern across a population, and answer questions not previously possible.

These data are created in the delivery of national public services and their use in research is secondary. As each data point is an experience or event in the life of a person, it is vital to obtain the views and opinions of publics if we are to maintain a social license to access and use this data.

Northern Ireland is currently the only of the three devolved jurisdictions of the UK (Scotland, Wales and Northern Ireland) that does not have a public panel to consider public data questions, including the secondary use of data, data research questions of public importance, data legislation, and other issues. While our data and research infrastructures develop, it is crucial that our public engagement infrastructure and processes do as well.

Only with both of these in place will the data culture in Northern Ireland shift from a closed, conservative one to a landscape that acknowledges the power of evidence-led knowledge creation, understands the key importance that publics play in providing and maintaining acceptability of data use, and promotes the use of data in research and development, decision and policy-making, and public services.

This paper will explore the rationale for and findings from a pilot to set up a public data panel unique in its approach, for the people of Northern Ireland, outlining success and challenges, and potential pathways forward.

